# A Python-based educational software tool for visualizing bioinformatics alignment algorithms

**DOI:** 10.5808/gi.22055

**Published:** 2023-03-31

**Authors:** Elis Khatizah, Hee-Jo Nam, Hyun-Seok Park

**Affiliations:** 1 Bioinformatics Laboratory, ELTEC College of Engineering, Ewha Womans University, Seoul 03760, Korea; 2 Center for Convergence Research of Advanced Technologies, Ewha Womans University, Seoul 03760, Korea

**Keywords:** educational software, Python, sequence alignment

## Abstract

Bioinformatics education can be defined as the teaching and learning of how to use software tools, along with mathematical and statistical analysis, to solve biological problems. Although many resources are available, most students still struggle to understand even the simplest sequence alignment algorithms. Applying visualizations to these topics benefits both lecturers and students. Unfortunately, educational software for visualizing step-by-step processes in the user experience of sequence alignment algorithms is rare. In this article, an educational visualization tool for biological sequence alignment is presented, and the source code is released in order to encourage the collaborative power of open-source software, with the expectation of further contributions from the community in the future. Two different modules are integrated to enable a student to investigate the characteristics of alignment algorithms.

## Introduction

What are the most crucial bioinformatics algorithms every bioinformatics student should know about? It could be useful to understand the principles of Bayesian and maximum likelihood tree construction. Alternatively, learning about homology modeling could be important. Perhaps, students must understand basic global, local, and multiple sequence alignments. In the end, this choice depends on the field of study, since bioinformatics is a broad term encompassing many different areas and methods of research.

Nonetheless, many people would agree that the most important algorithms to understand are basic sequence alignment algorithms. Sequence similarity is what we use as an approximation for biological and functional similarity, and alignment is the way to measure it. If one learns the underlying dynamic programming algorithm, then all the different classes of alignment become simple modifications of the parameters and edge cases of that algorithm.

Although abundant learning resources are available, most students still struggle to understand even the simplest sequence alignment algorithms. Applying visualizations to these algorithmic processes benefits both lecturers and students. Unfortunately, educational software for visualizing step-by-step processes in the user experience is rare. Visualizations of various types can help researchers and learners to achieve their goals. We observed that implementing visual learning materials significantly improved students’ performance.

There are stand-alone educational platforms that host numerous tools and databases for bioinformatics research and allow training to take place in a controlled environment, such as Geneious Basic, The BioKit, and OCRA [1-6]. However, our relatively narrow-scope project can be viewed as a detailed component of such platforms in a visual mode—specifically, we aimed to develop a step-by-step visualization of the processes involved in the Needleman-Wunsch (NW) and Smith-Waterman (SW) algorithms. Sequence alignment is the procedure of comparing two or more sequences by searching for a series of individual characters or character patterns that are in the same order in the sequence [7]. Both algorithms generate an alignment starting at the ends of two sequences by following a scoring scheme for matches, mismatches, and gaps. This mathematical procedure generates a matrix of numbers representing all possible alignments between the sequences, where the highest set of sequential scores defines an optimal alignment. Due to the large number of computational steps, the NW and SW algorithms are difficult for students when manually aligning long sequences. Our educational software has been developed as a complement to lectures on basic bioinformatics algorithms to help students understand this time-consuming process. This tool with a graphical user interface (GUI) will be beneficial for investigating how the algorithm works step by step. The results of our practices on different subjects demonstrated that students achieved better performance on exams when visual methods were used during the learning process.

## Implementation Details: The Step-by-Step Visualization Process of the NW-SW Algorithms

We implemented a visualization tool for two alignment algorithms with the use of TKinter, an official Python library used with the Tk GUI toolkit. This Python implementation is potentially beneficial for further development since Python is becoming the most well-suited programming language for bioinformatics and deep learning applications [8].

**Fig. 1.**
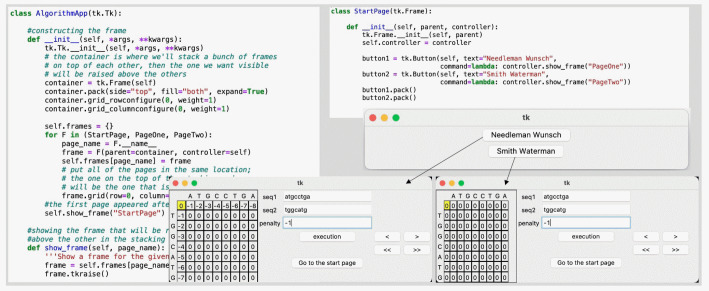
An exemplary code for arranging stacking frames toward the graphical user interface of Needleman-Wunsch and Smith-Waterman algorithms.

There are four phases in the implementation: matrix initialization, similarity score calculation, traceback and result generation [9]. As shown in Fig. 1, the main Python script arranges a user interface for multiple emerging frames. First and foremost, users can choose whether to apply the NW or SW method to align the sequences by clicking radio buttons labeled with the name of the algorithm. The selected method’s window then displays entry fields where users can manually input two pairs of sequences and a penalty gap value.

The scoring process begins by clicking the “execution” and “next” button tabs consecutively. An (*m*+1)×(*n*+1) zero matrix appears next to entry fields, with *m* and *n* indicating the length of *sequence 1* and *sequence 2*, respectively. After clicking the “execution” tab, the first row and the first column of the matrix score are filled with initial values that play the role of a boundary condition. The value in this boundary condition is the first difference between the NW and SW algorithms; the former uses a sequence number depending on the penalty gap value, while the latter simply makes all values zero.

Repeatedly clicking the next button, “>”, alters the score of each matrix’s element. Here, both algorithms use a similar scoring scheme for matches, mismatches, and gaps such that each entry in the matrix *D*(*i,j*) will be scored row by row recursively, subject to a boundary condition.


(1) $$D(i,j)=\max\left\{\begin{array}{l} D(i-1,\;j-1)+s(x_{i},\;y_{i})\\ D(i-1,j)+g\\ D(i,j-1)+g \end{array}\right.$$

**Fig. 2.**
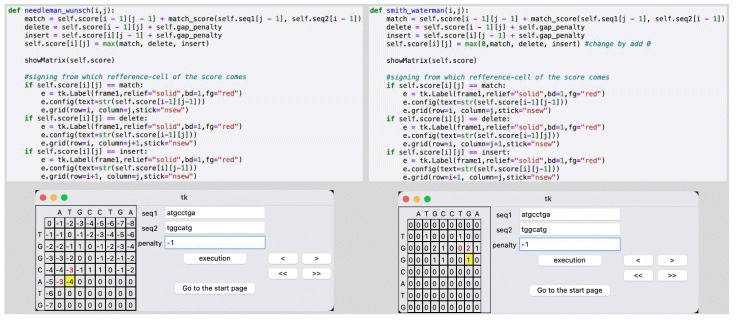
An exemplary code for similarity score calculation. The latest score with a yellow background in the graphical user interface refers to the nearest red-value element.

In equation, *s*(*i,j*) is the substitution score for residues *i* and *j*, and *g* is the gap penalty with *i* = 1, 2, 3, …, *m*+1 and *j* = 1, 2, 3, …, *n*+1. The similarity score calculation matrix up to a certain number of steps can be seen in Fig. 2. The latest score, on a yellow background, is displayed together with the nearest numbers (shown in red) that notate the elements that the score refers to. In addition, clicking the back button, “<”, brings users to the previous scored element, furnishing an easy way to glimpse the prior calculation.

**Fig. 3.**
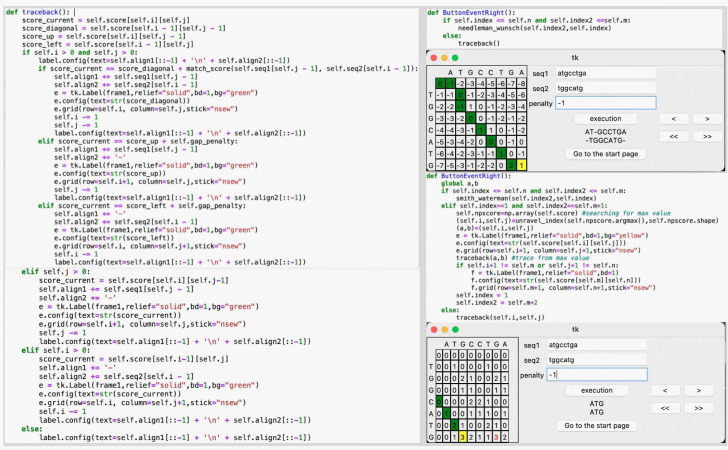
An exemplary code for traceback which generates alignment for both Needleman-Wunsch and Smith-Waterman algorithms.

The user continuously clicks the next button until the last element starts the traceback process, visualized along the elements in a green background color. Traceback is a process to find the highest set of sequential scores, which defines an optimal alignment. The initial value for this process is the second difference between the NW and SW algorithms. As shown in Fig. 3, the traceback process of the NW algorithm starts from the last matrix’s element, while for the SW algorithm, we must first find the maximum element’s value as a starting point. Immediately after the traceback process is complete, alignment outputs are finally displayed in the same frame where the input and controller buttons are located. In addition, for the user’s convenience, there are also “>>” and “<<” buttons to run all the forward and backward steps, respectively.

Overall, users can follow the alignment process step by step, giving them a real experience of applying the NW and SW algorithms, as well as writing it in their own pen-and-paper notes. We have conducted several tests to demonstrate this educational tool’s performance.

## References

[1] Magana AJ, Taleyarkhan M, Alvarado DR, Kane M, Springer J, Clase K (2014). A survey of scholarly literature describing the field of bioinformatics education and bioinformatics educational research. CBE Life Sci Educ.

[2] Oliver J, Pisano ME, Alonso T, Roca P (2005). The Web as an educational tool for/in learning/teaching bioinformatics statistics. Med Inform Internet Med.

[3] Kearse M, Moir R, Wilson A, Stones-Havas S, Cheung M, Sturrock S (2012). Geneious Basic: an integrated and extendable desktop software platform for the organization and analysis of sequence data. Bioinformatics.

[4] Hernandez-de-Diego R, de Villiers EP, Klingstrom T, Gourle H, Conesa A, Bongcam-Rudloff E (2017). The eBioKit, a stand-alone educational platform for bioinformatics. PLoS Comput Biol.

[5] Jackman SD, Mozgacheva T, Chen S, O'Huiginn B, Bailey L, Birol I (2019). ORCA: a comprehensive bioinformatics container environment for education and research. Bioinformatics.

[6] http://baba.sourceforge.net/.

[7] Mount DW (2001). Bioinformatics: Sequence and Genome Analysis.

[8] Dhruv AJ, Patel R, Doshi N Python: the most advanced programming language for computer science applications.

[9] Xu X, Chan Y, Xu K, Zhang J, Wang X, Yin Z SLPal: Accelerating long sequence alignment on many-core and multi-core architectures.

